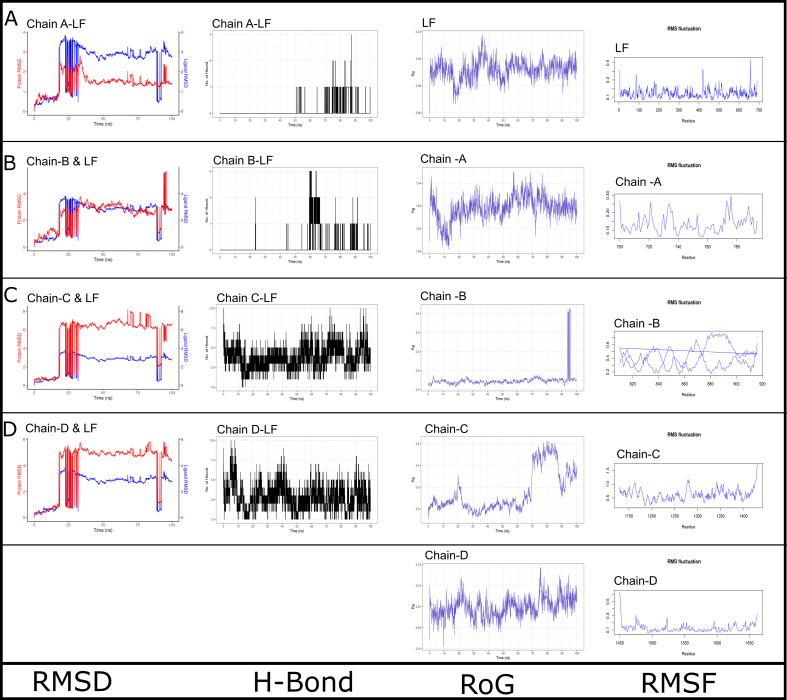# Lactoferrin's role in modulating NF-κB pathway to alleviate diabetes-associated inflammation: A novel *in-silico* study

**DOI:** 10.1016/j.heliyon.2024.e34051

**Published:** 2024-07-03

**Authors:** Muhammad Asif Arain, Gul Bahar Khaskheli, Ghulam Shabir Barham, Illahi Bakhsh Marghazani

**Affiliations:** a Faculty of Animal Husbandry & Veterinary Sciences, Sindh Agriculture University, Tandojam, 70060, Pakistan; b Faculty of Veterinary and Animal Sciences, Lasbela University of Agriculture Water and Marine Sciences, Uthal, 90150, Pakistan

**Keywords:** Lactoferrin, Anti-inflammatory, Immune-modulatory, NF-κB pathway, Molecular docking and simulation, In silico approach

## Abstract

Lactoferrin (LF), a multifunctional glycoprotein found in mammalian milk and various exocrine secretions, plays a pivotal role in modulating various responses. Lactoferrin plays a significant role in type-2 diabetes by improving hepatic insulin resistance and pancreatic dysfunction however, the exact mechanism for this improvement is not thoroughly elucidated. To this date, there are no evidence that attributes the direct interaction of lactoferrin with components of NF-κB pathway. Considering this precedent, the current study aimed to investigate the interaction of LF with key components of NF-κB pathway using molecular docking and simulation approaches. Results indicated that LF has shown highly stable interactions with IL-1β, IL-6, IκBα and NF-κB, and relatively weaker interactions with IKK and TNF-α. All four trajectories, including root mean square of deviations (RMSD), root mean square of fluctuation (RMSF), hydrogen bond interactions, and radius of gyration (RoG), confirmed the stable interactions of LF with NF-κB pathway components. Molecular Mechanics/Generalized Born Surface Area (MM/GBSA) analysis further supports their stable interactions. To the best of our knowledge, this is the first study to provide convincing evidence that LF can interact with all six major components of the NF-κB pathway. This study provides pioneering in-silico evidence that lactoferrin (LF) can interact with all six major components of the NF-κB pathway, demonstrating highly stable interactions with IL-1β, IL-6, IκBα, and NF-κB, and relatively weaker interactions with IKK and TNF-α. These findings suggest that LF and its peptides have significant potential for both preventive and therapeutic applications by targeting the NF-κB pathway to inhibit inflammation, thereby improving insulin sensitivity and aiding in the management of diabetes.

## Highlights

• Lactoferrin (LF) is a versatile glycoprotein found in mammalian milk and various exocrine secretions.
• LF and its peptides may be used in preventive and treatment strategies by targeting the NF-κB pathway.
• Modulating the NF-κB pathway can reduce inflammation, improve insulin sensitivity, and enhance insulin signaling and glucose uptake.
• LF showed highly stable interactions with IL-1β, IL-6, IκBα, and NF-κB. while relatively weaker interactions with IKK and TNF-α.
• LF improves hepatic insulin resistance and improves pancreatic function for batter glycemic control.

## 1 Introduction

Lactoferrin (LF) is an antimicrobial, iron-binding mammalian glycoprotein of the transferrin family secreted by polymorphonuclear leukocytes and exocrine glands, in milk and at the mucosal surface [1,2]. Abundant expression and secretion of lactoferrin, particularly in milk and fluids of the digestive tract, are related to its implication in the first line of host defence [3,4]. It has antimicrobial efficacy against various pathogens, such as bacteria, fungi, viruses, and parasites [5,6]. The antimicrobial effect of LF has been mainly attributed to its capacity to deprive iron from bacteria [[7], [8], [9]]. LF binds and sequesters iron and also interacts with lipoproteins, apolipoproteins, proteoglycans, lymphocytes, enterocytes, and nucleolins [3]. Lactoferrin is an 80-kDa, iron-binding glycoprotein present in milk and exocrine fluids. It consists of a single-chain polypeptide with two globular lobes and is relatively resistant to proteolysis. The N-lobe is divided into N1 and N2, and the C-lobe is divided into C1 and C2. The iron-binding cleft is situated between the domains in each lobe [10]. As an integral part of the innate immune system, LF is a well-known immunomodulator of leukocyte populations that performs its regulatory function by inhibiting several cytokines [11,12]. Lactoferrin, a multifunctional glycoprotein, holds considerable promise in the therapeutic landscape of diabetes mellitus (DM). Its presence serves as a notable indicator of type 2 diabetes mellitus (T2DM), owing to its anti-inflammatory properties and its role in the downregulation of pro-inflammatory mediators. The decline in lactoferrin levels observed in T2DM may exacerbate the inflammatory milieu, consequently augmenting the levels of inflammatory markers associated with heightened inflammatory activity. However, the increased understanding of link between LF and management of T2DM is not thoroughly clear. Lactoferrin affects the inflammatory mechanisms mainly affecting nuclear factor-kappa B (NFκB) signalling pathway.

The NFκB signalling pathway is vital for regulating the immune response, inflammation, and various cellular processes. While its activation is necessary for initiating the body's defence mechanisms, prolonged or excessive activation can lead to chronic inflammation, cancer, and autoimmune diseases. Therefore, suppressing the NF-κB pathway can offer several potential benefits, including preventing age-related inflammation, insulin resistance, and various inflammatory conditions [13,14]. Additionally, inhibiting NF-κB may aid in combating cancer development, heart muscle changes, asthma, and impaired wound healing. However, it's important to regulate the inhibition of NF-κB carefully, as excessive or prolonged inhibition can have harmful effects, such as long-term suppression of the immune system. Furthermore, targeting specific components of the NF-κB pathway involved in particular diseases may help minimize systemic toxicity and avoid broad suppression of the body's innate immunity [13]. In total there are three major components of NF-κB pathway 1) signalling cytokines (TNF-α, IL-1β, IL-16), 2) IKK-β (activation enzyme) 3) IκB-α (keeps NF-κB inactive) 4) NF-κB complex protein. Recent metanalysis revealed that LF affects the upstream and downstream components of NF-κB pathway by reducing TNF-α, IL-1β, IL-6 cytokines level and IKK-β, IKB-α and NF-κB protein (Fig. 1). However, the effect of LF on NF-κB pathway is still unknown. To this date, there is no evidence reported for the interaction NF-κB for LF. Here we utilized the extensive molecular dynamics and simulation approach that provide evidence of significant inhibition of components of NF-κB pathway by lactoferrin.

**Fig. 1:**
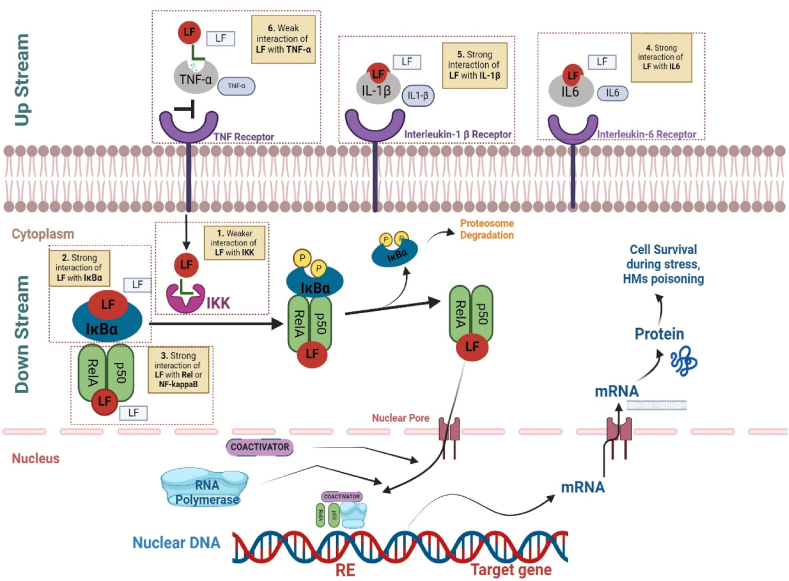
Schematic representation of Lactoferrin's Interaction with the NF-κB Pathway. Lactoferrin (LF) has shown stable interactions with IL-6 (1), IL-1β (2), IκBα (3), and NF-κB (4) and relatively weaker interactions with IKK (5) and TNF-α (6). This figure illustrates the molecular docking and simulation results, highlighting the stable binding of LF with key components of the NF-κB pathway, which plays a crucial role in regulating immune responses and inflammation. The stable interactions with IL-6, IL-1β, IκBα, and NF-κB suggest that LF can effectively modulate the NF-κB pathway, potentially reducing inflammation and improving insulin sensitivity, which is significant for managing diabetes-associated inflammatory syndrome. The relatively weaker interactions with IKK and TNF-α indicate that while LF can interact with these components, the binding is less stable compared to the other component.

## 2 Material and methods

### 2.1 Molecular structure models

The native structure of camel Lactoferrin was retrieved from the PDB (entry 1DTZ). Well resolved structures of upstream components including three TNF-α, IL-6 and IL-1β was acquired from PDB. Besides, resolved structure through x-ray crystallography of downstream components IKK‐β, IκB, and NF‐κB (p65) were also acquired. Details of each structure are provided below. TNF‐α, IL1-β, and IL‐6 protein structures are downloaded from the Protein Data Bank (PDB) [15]. Structures having higher resolutions, low R values, stable conformational state were selected from PDB repository details of their resolution are provided at individual level. The 3D crystal structure of TNF-α (1tnf) has a 2.60 Å resolution that consists of three chains (A, B, and C) [16]. IL1-B with (4DEP) has a 3.10 Å resolution that consists of six chains (A, B, C,D,E,F) [17]. Interleukin-6 with (IL-6) consists of a single chain (A) [18]. The 3D crystal structure of IKB-α (1NFI) having a 2.70 Å of resolution that consist of six chains (A, B, C,D, E, F) [19], structure of IKK with (4KIK) having a 2.83 Å of resolution that consist of two chains (A, B) [20], structure of NFKB (1NFK) having a 2.30 Å of resolution that consist of four chains (A, B, C,D) [21]. Structure of LF (IKB-α) having a 2.40 Å of resolution that consist of a chain (A) [22]. All the 3D protein structures were obtained in pdb format from the Research Collaboratory for Structural Bioinformatics (RCSB).

### 2.2 Molecular docking

The interaction between proteins was studied using a molecular docking tool, AutoDock Vina [23]. The center coordinates (x, y, z) of binding sites were acquired through Discovery Studio Visualizer as (35.18, 44.25, −10.96). The dimensions of the grid box were set as X: 26, Y:26, and Z:26 (unit of dimensions is Å). The number of binding modes that could be generated was limited to 20. Moreover, the maximum energy difference between the best and worst binding modes was set to 4. Validation of docking protocol was done by performing the docking of the co-crystallized ligand at the active site of the protein. Therefore, docking was performed with reference molecule, and the RMSD value between experimental and docked reference was calculated to validate the docking protocol. The analysis of docking result was carried out using Discovery Studio v19.1.0.18287.

### 2.3 Molecular dynamics simulations

The complexes CLF-TNF-α, CLF- IL-1β and CLF-IL-1β were studied by molecular dynamics simulation with the GROMACS and CHARMM27 force field [24]. Proteins were solvated in a cubic box using the TIP3P water model. To neutralize the overall charge of the systems, Na and Cl ions were added as appropriate. Periodic boundary conditions were applied from this step onward. The system was first energy minimized using the steepest descent algorithm to relax high-energy contacts. After energy minimization, the system was simulated under the NPT ensemble for 500 ps, with initial velocities taken from a Maxwell-Boltzmann distribution corresponding to 100 K. During this initial simulation time, the peptide and DNA atoms were positionally restrained while the temperature was gradually increased from 100 K to 300 K at 1 atm. Bond lengths were constrained for all atoms using the LINCS algorithm (SETTLE for water), allowing a time step in the leap-frog integrator of 2 fs. Temperature and pressure were couple to the reference values using the Nosé-Hoover and Parrinello-Rahman algorithms, respectively. Additional 100 ps at 300 K and 1 atm, without position-restraints, were subsequently run. In the production phase, the equilibrated systems were run in the NPT ensemble at 1 atm and 300 K for 200 ns. Long-range electrostatics were evaluated using the Particle Mesh Ewald (PME) algorithm. The real space component of PME and the van der Waals interactions were calculated with a cutoff of 1.0 nm. Three replicates of 200 ns were run per system, with different initial configurations generated by insertion of the peptides at random positions. Dynamics and stability of each protein-protein interaction including root mean square deviation (RMSD), root-mean-square-fluctuations (RMSF), solvent accessible surface area (SASA), contacting surface area (CSA), hydrogen bonds, salt bridges, and center of mass distance were analyzed during the simulation using GROMACS built-in tools. An RMSD-based conformational clustering algorithm, using the gmx-cluster module of GROMACS, was applied to extract representative structures. The clusters were obtained using a cut-off of 1.5 Å for the RMSD to the centroid.

### 2.4 Binding free energy estimates

Binding free energies were estimated CLF-TNF-α, CLF-IL-6 and CLF-IL-1β complexes using molecular mechanics energies in combination with Poisson-Boltzmann and surface area continuum solvation (MM/PBSA). The calculations were performed with the g_mmpbsa program (https://rashmikumari.github.io/g_mmpbsa/) [25], using the single trajectory approach. The solute dielectric constant was set to 8 [26] and the ionic strength was chosen to correspond to a NaCl concentration of 150 mM. The calculation of the G_polar_ solvation term was performed with the linearized Poisson-Boltzmann (PB) equation using a grid resolution of 0.05 nm and the *bondi* set of atomic radii. The G_nonpolar_ term was calculated with the SASA model using default parameters. The entropic component of the binding free energy was disregarded. The average binding energy and its standard deviation were calculated with the MmPbSaStat.py python script (http://rashmikumari.github.io/g_mmpbsa/) using the second half of the simulations production phase (100–200 ns), by taking 1000 snapshots at 100-ps intervals. To estimate the contribution of each residue to the total binding free energy, the MmPbSaDecomp.py python script was used. It should be noted that this approach represents a crude estimate of the binding free energy that, most certainly, severely overestimates the real value, as noted by several authors. However, the limitations of the approach are likely to affect the related systems studied here in similar ways and are therefore expected to allow for a qualitative comparison.

### 2.5 Free energy landscape (FEL)

A free energy landscape (FEL) approach was used to determine the lowest energy state stable conformation. The boundaries between the subspaces represent the intermediate conformations. The FEL was determined by using the g_sham module integrated into Gromacs. The FEL was calculated from the following equation using the first two PCs:

C2) ΔG(PC1,PC2)=KBInP(PC1,P

## 3 Results

### 3.1 Evaluating interaction of downstream components with lactoferrin

#### 3.1.1 Verification of docking and simulation parameters and interaction of LF with IKK

**Fig. 2:**
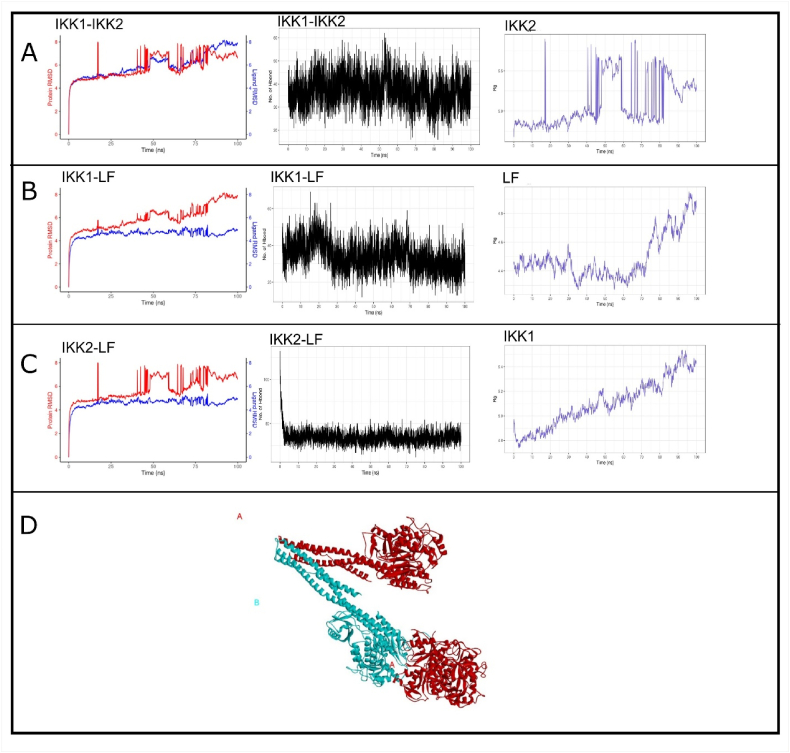
Two chains IKK1 and IKK2 have shown stable interaction, Panel A shows IKK1-IKK2 RMSD plot, H-bond interaction and RoG plot of IKK2. Panel B indicates IKK1-LF RMSD plot with its H-bond interaction and RoG plot. Panel C shows the interaction of IKK2 and LF with H-bond interaction and RoG plot. Panel D shows dual interaction of lactoferrin with IKK2 chain.

The IKK (IκB kinase) complex is a central regulator of the NF-κB signaling pathway, which is essential for the plethora of functions attributed to NF-κB. The IKK complex consists of two catalytic subunits, IKK1 (also known as IKKα) and IKK2 (also known as IKKβ), and a regulatory subunit, IKKγ (also known as NEMO). The IKK complex is activated by phosphorylation of specific serine residues within the activation loop of IKK1 and IKK2, which renders the kinase domain catalytically active. To verify the docking and simulation parameters a blind docking was performed for both chains (IKK1 and IKK2: Table 1). This interaction was evaluated at an atomistic level for up to 100ns, with the root mean square of deviation (RMSD) showing strong convergence between the trajectories of both IKK1 and IKK2 (Fig. 2A). We further consulted the H-bond spectra that suggested the presence of on average 40 H-bonds during 100ns. We further evaluated the compactness of each chain during simulation by radius of gyration (RoG) trajectory. IKK1 have shown consistent increase in the RoG however IKK2 have shown sharp decrease and increase in radii (Fig. 2A). Considering the strong convergence of both proteins and presence of 40 H-bond interaction warrants the authenticity of docking and simulation parameters. We further performed simulation of each chain of IKK with lactoferrin. Upon molecular simulation trajectories analysis, RMSD plot have suggest that chain IKK1 have shown initial congruence however after 80ns the deviation between both proteins have significantly increased by more than 2 Å. However, till the end of simulation 30 H-bond interactions were stable linking both proteins (Fig. 2B). In-terms of molecular compactness, Lactoferrin have shown increase in flexibility by 0.2 Å. Contrast to IKK1, simulation with IKK2 (IKKβ) suggest congruence of both protein trajectories with deviation of less than 2 Å and average 40 H-bond interactions. Deviations for short periods in the IKK2 trajectories were also observed between 50 and 60 ns (Fig. 2C). Considering overall congruence and significant number of H-bond interactions between IKK2 and lactoferrin strongly endorse their stable interaction. Docking analysis suggest that IKK2 successfully docked with lactoferrin at more than one site of lobe C1 (Fig. 2D, lactoferrin indicated in RED and IKK2 in blue). Metanalysis also confirms that reduction of IKK (7.37 fold) were observed upon exposure to lactoferrin in 25 selected studies with no significant heterogeneity in data. Following in-vivo and molecular dynamics confirmation it's been suggested that lactoferrin lobe C1 has significant immunogenic properties that can allow direct interaction with IKK.

**Table 1:** Molecular interactions of IKK with Lactoferrin.

Complex	Hydrogen Bonds	Hydrophobic Interactions
IKK_LF (Chain A-B)	Asp493(B), Ser489(B), Glu495(B), Glu499(B), Lys659(B), Gln477(B), Ser474(B), Gln478(B), Asp484(B), Gln651(B), Trp655(B), Lys482(B), Trp655(A), Gln651(A), Lys652(A), Lys659(A), Glu499(A), Ser474(A), Gln477(A), Lys480(A), Lys482(A), Ser489(A), Asp493(A), Asp484(A)	Asp493(B), Ser489(B), Glu495(B), Glu499(B), Lys659(B), Gln477(B), Ser474(B), Gln478(B), Asp484(B), Gln651(B), Trp655(B), Lys482(B), Trp655(A), Gln651(A), Lys652(A), Lys659(A), Glu499(A), Ser474(A), Gln477(A), Lys480(A), Lys482(A), Ser489(A), Asp493(A), Asp484(A)
IKK_LF(chain B-A)	Asp217(A), Glu223(A), Arg200(A), Glu226(A), Ser293(A), Ala222(A), Asp220(A), Glu85(A), Glu221(A), Lys197(A), Ser303(A), Arg24(A), Gly290(A), Gln21(A), Gln287(A), Ser219(A), Glu216(A), Arg47(B), Asn54(B), Ser51(B), Arg53(B), Gly27(B), Cys46(B), Gln45(B), Arg57(B), Arg579(B), Phe182(B), Glu49(B), Gly25(B), Thr9(B), Arg20(B), Gly22(B)	Tyr227(A), Gln295(A), Lys296(A), Ser291(A), Pro292(A), Phe20(A), Cys(A), Ser35(A), Gly294(A), Gln23(A), Arg27(A), Asp302(A), Lys285(A), Pro284(A), Asp281(A), Phe286(A), Phe26(B), Ile30(B), Asn28(B), Val183(B), Ser4(B), Trp3(B), Pro88(B), Gln48(B), Leu50(B), Leu7(B), Thr8(B), Glu19(B), Thr23(B), Tyr169(B)

### 3.2 Interaction of lactoferrin with IκBα

**Fig. 3:**
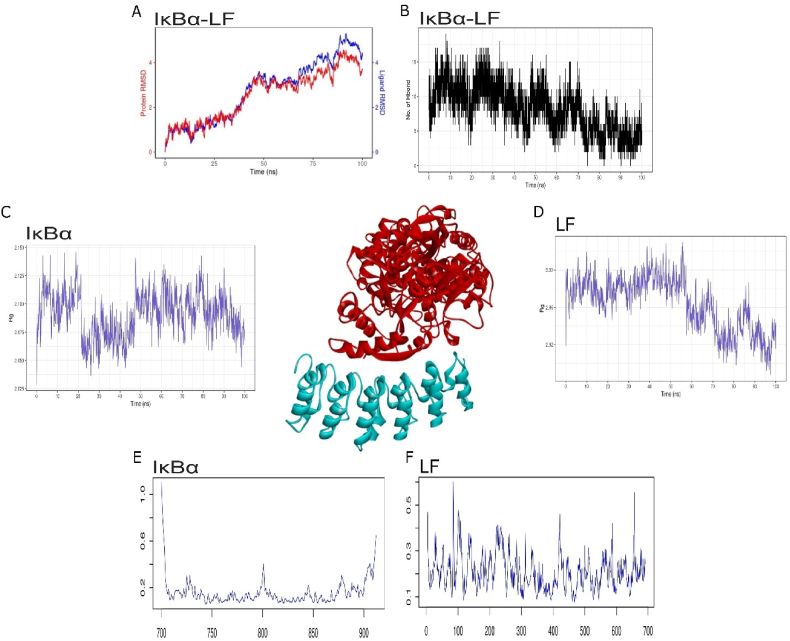
Molecular interaction of Lactoferrin (red) and IκBα (blue). Molecular dynamics simulation of 100ns between Lactoferrin and IκBα used to traced A) RMSD trajectory, B) H-bond interaction, C) RoG plot of IκBα, D) RoG plot lactoferrin, E) RMSF plot of IκBα (200 a. ã700–900) and F) RMSF plot of Lactoferrin (1–700 a.a). (For interpretation of the references to color in this figure legend, the reader is referred to the Web version of this article.)

IκBα plays a crucial role in the NF-κB signalling pathway by sequestering NF-κB in the cytoplasm. This prevents its translocation into the nucleus and subsequent activation of NF-κB target genes. The structure of IκBα consists of ankyrin repeat domains, which are responsible for its interaction with NF-κB. The six ankyrin repeat domains stack together and interact with the C-terminal domains of NF-κB, which conceals its nuclear localization signals and promotes its cytoplasmic retention. This interaction is crucial for the transcriptional inactivation of NF-κB. IκBα acts as a negative regulator of the NF-κB pathway, and its structural features are critical for tightly controlling NF-κB activity in the cell. The process of blind docking IκBα and LF was carried out, with a two-dimensional interaction analysis suggesting the presence of 18 hydrogen bonds and 8 hydrophobic interactions between LF and IκBα (Table 2: Fig. 3). The alpha helical structure of the N1 domain of LF, specifically between residue 23–53, was found to have interacted with IκBα (Fig. 3). This interaction was evaluated at an atomistic level for up to 100ns, with the RMSD showing strong convergence between the trajectories of both IκBα and LF. Initially, both molecular trajectories showed a deviation of less than 0.1 Å until 70 ns (Fig. 3A). The trajectory of H-bonds provides evidence of a minimum of 10 H-bond interactions that stabilized this p-p interaction until 70 ns. An increase in deviation of up to 0.2 Å was observed at the end of the simulation, probably due to the gradual reduction of H-bond interactions (Fig. 3B). Corresponding trajectories of RMSD and H-bond have confirmed the stable interaction between IκBα and lactoferrin. The tight packing of the protein influences protein dynamics and structural stability [27]. The structural compactness of lactoferrin and IκBα was evaluated using simulation trajectories, specifically the Radius of Gyration (RoG). The RoG spectrum for lactoferrin showed a gradual decrease in radius from 3 to 2.92 Å, while the RoG trajectory demonstrated an overall average of 2.05 Å, indicating stability and compact protein-protein interaction (Fig. 3C and D). The effect of lactoferrin binding on IκBα was also studied using the RMSF trajectory. The average RMSF value remained below 0.2 Å, with only one small peak of fluctuation observed at 100th residue, marking the end of the second ankyrin domain (Fig. 3 E and F). Overall, the protein exhibited stable and less flexible changes throughout the simulation. All four trajectories confirmed the stable interaction between IκBα and lactoferrin, consistent with a meta-analysis of 25 case studies. Upon exposure to lactoferrin, a decrease of up to 15.02-fold was observed in the levels of IκBα compared to the LPS-exposed group (95 % CI, −20.44 to −9.6; Z = 5.43; p < 0.00001) [28].

**Table 2:** Molecular interactions of IκBα with Lactoferrin.

Complex	Hydrogen Bonds	Hydrophobic Interections
IKB alpha_LF (Chain A-F)	Asn52(A), Gln44(A), Arg39(A), Arg53(A), Glu51(A), Gln47(A), Arg24(A), Gln23(A), Arg30(A), Ser35(A), Ly28(A), Arg27(A), Gln112(F), Asn182(F), Leu227(F), Ile192(F), Glu213(F), Arg218(F), Asp226(F), Asn122(F), Gln154(F), Glu153(F), Asp75(F), Asp73(F), Arg143(F), Glu85(F), Ile83(F)	Pro33(A), Cys36(A), Lys38(A), Gln7(A), Ile37(A), Phe20(A), Ser5(A), Phe142(F), Thr121(F), His193(F), Leu223(F), Arg260(F), Tyr195(F), Ile120(F), Leu110(F), Leu117(F)

### 3.3 Interaction with NF-κB

**Fig. 4:**
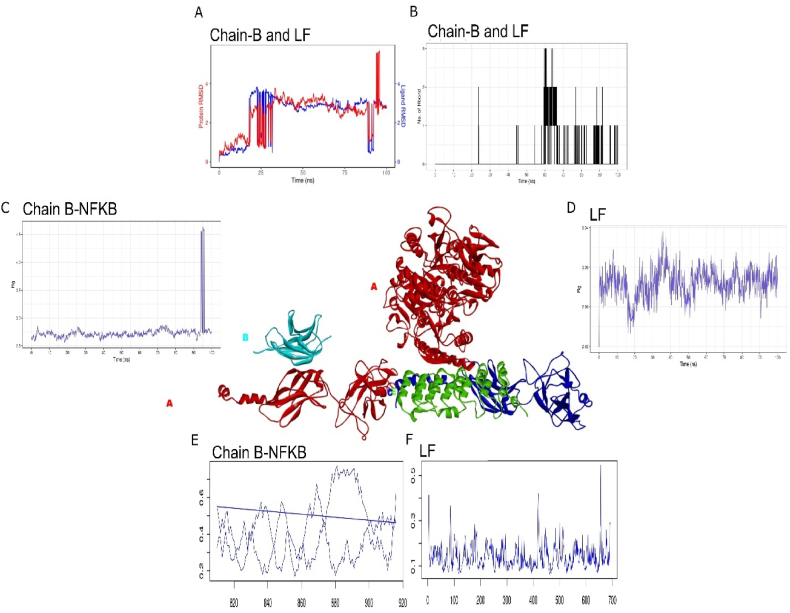
Molecular interaction of lactoferrin (in red color) with three chains B (in blue color), C (green) and E (in dark blue color). Molecular dynamics simulation of 100ns between Lactoferrin and Chain b used to traced A) RMSD trajectory, B) H-bond interaction, C) RoG plot of B chain, D) RoG plot lactoferrin, E) RMSF plot of B chain (120 a.ã800–920) and F) RMSF plot of Lactoferrin (1–700 a.a). (For interpretation of the references to color in this figure legend, the reader is referred to the Web version of this article.)

Functionally, NF-κB is a key regulator of inflammatory responses and is involved in the pathogenesis of various diseases, including cancer, arthritis, chronic inflammation, and asthma. The activation of NF-κB is tightly regulated through upstream signaling molecules and downstream feedback loops. Overall, NF-κB plays a critical role in the immune system and the development of inflammatory diseases. It consist of four chains A, B, C and D with length of 325 amino acid residue [21]. Details of their interactions are indicated in Table 3. Molecular dynamics suggest strongest interaction with chain B of NF-κB. Upon simulation of 100ns, trajectories of lactoferrin and chain B have shown highest degree of similarity with average deviation of less than 0.1 Å (Fig. 4A). However, reduced number of H-bond interactions were observed during the simulation of 100ns (Fig. 4B). RoG of lactoferrin have shown strong compactness whereas B chain have shown sharp flexibility at 95 ns which is also observed in RMSD plot (Fig. 4C and D). RMSF trajectories of NF-κB have also shown degree of fluctuations during the interaction with lactoferrin but fluctuation of 0.7 Å were observed (Fig. 4 E and F). Overall, RMSD, RoG and RMSF have shown evidence of stable interaction between NF-κB and lactoferrin. Metanalysis confirms that reduction of NF-κB (3.88 fold) were observed upon exposure to lactoferrin in 25 selected studies with no significant heterogeneity in data. Following in-vivo and molecular dynamics confirmation it's been suggested that lactoferrin has significant immunogenic properties that can allow direct interaction with NF- κB. MD simulations for all four chains are indicated in Fig. S1.

**Table 3:** Molecular interactions of three chains of NF-κB with Lactoferrin.

Complex	Hydrogen Bonds	Hydrophobic Interections
NFKB _LF (Chain A-B)	Asp835(B), Arg816(B), Asp818 (B), Tyr831(B), Glu829(B), Asp866(B), Arg869(B), Arg926(A), Asp923(A), Glu891(A), Asn880(A), Val879(A), Arg878(A), Asp897(A),	Phe 871(B), Cys 834(B), Val 815(B), Leu 833(B), Val874(B), His 868(B), His 925(A), Val931(A), Phe893(A), Leu895(A), Cys877(A), Val(A)
NFKB_LF (Chain A-C)	Lys1424(C), Phe1428(C), Ser1429(C), Pro848(A), Arg846(A), Glu783(A)	Met1423(C), Pro1427(C), Gly1430(C), Ser849(A), Gly850(A), Pro749(A), Pro786(A),
NFKB_LF (Chain A-E)	Glu1466(E), Gln1476(E), Glu1472(E), Arg1475(E), Lys1478(E), Arg851(A), Arg864(A), Thr751(A), Thr844(A), Glu781(A), Asp774(A)	Leu1469(E), Ala1468(E), Gly850(A), Pro852(A), Glu783(A)

### 3.4 Upstream effects of lactoferrin on NF-κB signalling pathway

#### 3.4.1 Interaction with human interleukin-6 (IL-6)

**Fig. 5:**
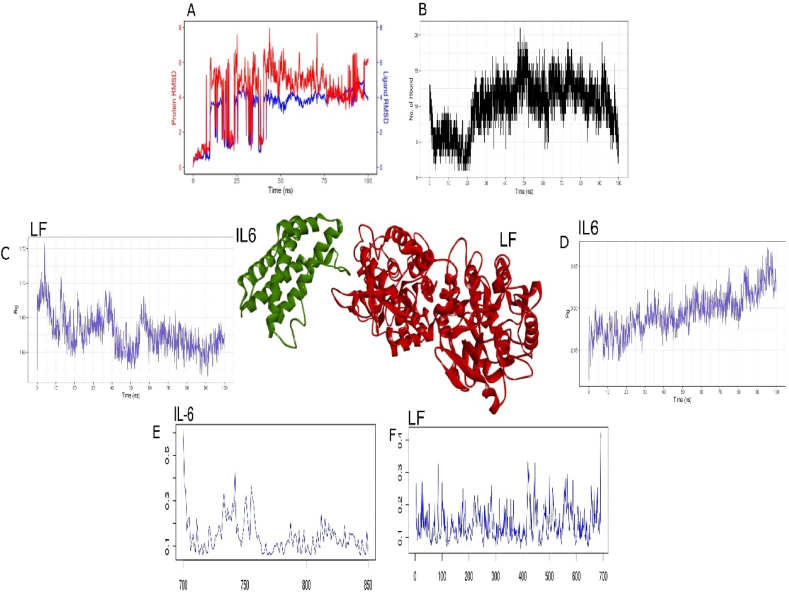
Molecular interaction of lactoferrin (in red color) with IL6 (in green color). Molecular dynamics simulation of 100ns between Lactoferrin and IL-6 used to traced A) RMSD trajectory, B) H-bond interaction, C) RoG plot of IL-6 chain, D) RoG plot of lactoferrin, E) RMSF plot of IL-6 (150 a.ã700–850) and F) RMSF plot of Lactoferrin (1–700 a.a). (For interpretation of the references to color in this figure legend, the reader is referred to the Web version of this article.)

The structure of human IL-6 consists of four alpha-helices and a four-stranded antiparallel beta-sheet arranged in a compact globular fold. The protein has a molecular weight of approximately 21 kDa and is composed of 184 amino acids. Blind docking of IL-6 and lactoferrin was performed. (Table 4). We further evaluate the stability of this complex by subjecting to MD simulation of 100ns. RMSD peak strongly suggest strong convergence of LF and IL-6 throughout the simulations (Fig. 5A). Sharp fluctuations were observed in the trajectory of LF-IL6 complex within 0–50ns. At the end of simulation consistent convergence of LF and IL-6 was achieved, and deviation was less than 0.5 Å. Initial, fluctuation can be attributed to reduced number of H-bond interaction (Fig. 5B). Gradual increase in H-bond interaction occurred till 50 ns with average 15 H-bond interactions between 50 and 100ns. Besides, hydrophobic interaction could also have played important role in stabilizing the LF-IL6 complex. The tight packing of the protein influences protein dynamics and structural stability [27]. We further evaluated the compactness of interleukin-6 and lactoferrin. Radius of gyration (RoG) have shown gradual increase in the flexibility of lactoferrin at end of simulation it reached up to 3.05 Å (Fig. 5D). However, IL-6 has shown gradual reduction in RoG of 1.72 to 1.66 Å from initial peak of 1.72 Å (Fig. 5C). Both trajectories of each protein have shown signatures of highly stable molecular interaction between lactoferrin and IL-6. We further evaluated the effect of IL-6 upon binding of lactoferrin using RMSF. This trajectory strongly suggests reduced and stable conformational changes in both proteins with average fluctuations did not cross the threshold of 0.3 Å (Fig. 5 E and F). Overall, all four interaction trajectories provide evidence about the stable interaction of IL-6 with lactoferrin. This stable interaction can be confirmed by evaluating 25 selected studies where downregulation of IL-6 cytokines were observed upon lactoferrin exposure as compared to LPS-exposed groups [28].

**Table 4:** Molecular interactions of IL-6 with Lactoferrin.

Complex	Hydrogen Bonds	Hydrophobic Interections
IL6_LF(Chain A-B)	Arg721(B), Glu732(B), Asn729(B), Glu736(B), Trp838(B), Asp422(A), Ser421(A), Lys416(A), Arg622(A), Asn623(A)	Lys735(B), Ala737(B), Leu738(B), Met730(B), Asn836(B), Thr647(A), Glu648(A), Cys427(A), Cys649(A), Cys627(A), Pro628(A), Asp629(A)

### 3.5 Interaction of lactoferrin with IL-1β

**Fig. 6:**
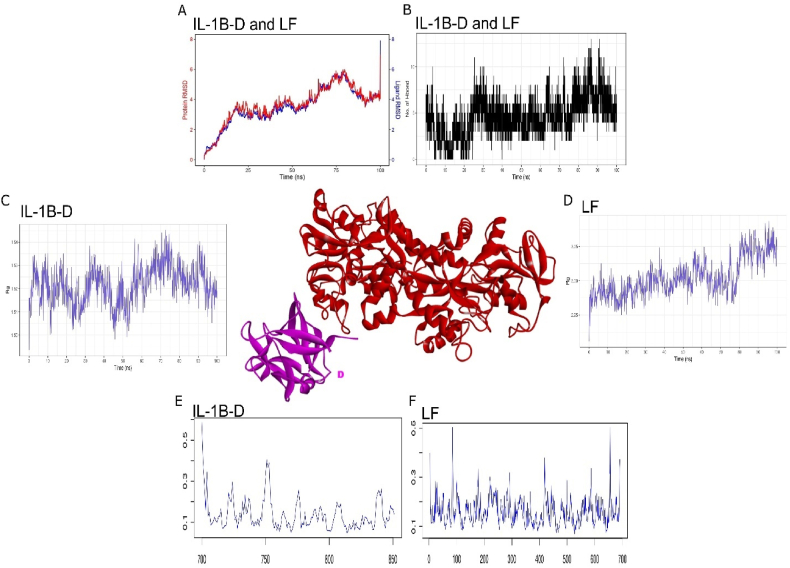
Molecular interaction of lactoferrin (in red color) with IL6 (in purple color). Molecular dynamics simulation of 100ns between Lactoferrin and IL-1β-D chain used to traced A) RMSD trajectory, B) H-bond interaction, C) RoG plot of IL-1β-D chian chain, D) RoG plot of lactoferrin, E) RMSF plot of IL-6 (150 a.ã700–850) and F) RMSF plot of Lactoferrin (1–700 a.a). (For interpretation of the references to color in this figure legend, the reader is referred to the Web version of this article.)

Interleukin-1β (IL-1β) is a therapeutic target due to its involvement in various pathological conditions, including inflammation, autoimmune diseases, and malignancies [29,30]. IL-1β is thought to play an important role in cancer invasiveness, progression, and metastasis, and its inhibition could potentially be a therapeutic strategy for certain malignancies [29]. Interleukin-1β have two chains (A, D) with 158 number of total amino acid residues. We performed the blind molecular docking of lactoferrin with A and D chain of IL-1β (Table 5). Lactoferrin N1 lobe including residue 139–171 have shown continuous H-bond and hydrophobic interaction with two different points (residues:1–4, 54, 93) of D-chain IL-1 β. In total 10 H-bond and 9 van Der Waals interactions were observed. Upon simulation of 100ns, chain D (IL-1β-D) have shown highly consistent interaction with lactoferrin. RMSD of both proteins have shown negligible deviations with less than <0.1 Å (Fig. 6A) and consistent increase in the number of H-bond interaction were also observed (Fig. 6B). Exceedingly congruent trajectories between both proteins were probably due to 10 stable H-bond interactions. RoG, which is a measure of compactness of interaction between both proteins have also shown slight increase in their radii. IL-1β-D have shown increase of 0.02 Å (Fig. 6C), whereas lactoferrin have shown increase of 0.1 Å (Fig. 6D). Furthermore, we also perform RMSF trajectory that suggest reduced and stable conformational changes in both proteins with average fluctuations did not cross the threshold of 0.4 Å (Fig. 6 E and F). Overall, all four trajectories provide evidence that Lactoferrin can form stable interaction with interleukin-1β using highly immunogenic N1 lobe. Metanalysis conducted on 25 selected studies have revealed lower levels of 1L-1β cytokine upon exposure to lactoferrin without significant heterogeneity [28]. Congruence in *In-silico* and *In-vivo* experiments strongly suggest that LF directly binds with interleukin-1β and cause inhibition of NF-κB signalling pathway.

**Table 5:** Molecular interactions of IL-1β with Lactoferrin.

Complex	Hydrogen Bonds	Hydrophobic Interections
ILIBd_LF(Chain A-D)	Arg171(A), Asp162(A), Gln165(A), Pro142(A), Asp54(D), Lys93(D), Ala1(D), Pro2(D), Arg4(D)	Arg151(A), Thr139(A), Asn168(A), Pro167(A), Gly164(A), Phe166(A), Ala147(A), Pro144(A), Glu143(A), Asn53(D), Glu105(D), Phe150(D),Ile56(D), Val47(D), Phe46(D), Val3(D),Ser5(D)

### 3.6 Evaluating the interaction of TNF-α and lactoferrin

**Fig. 7:**
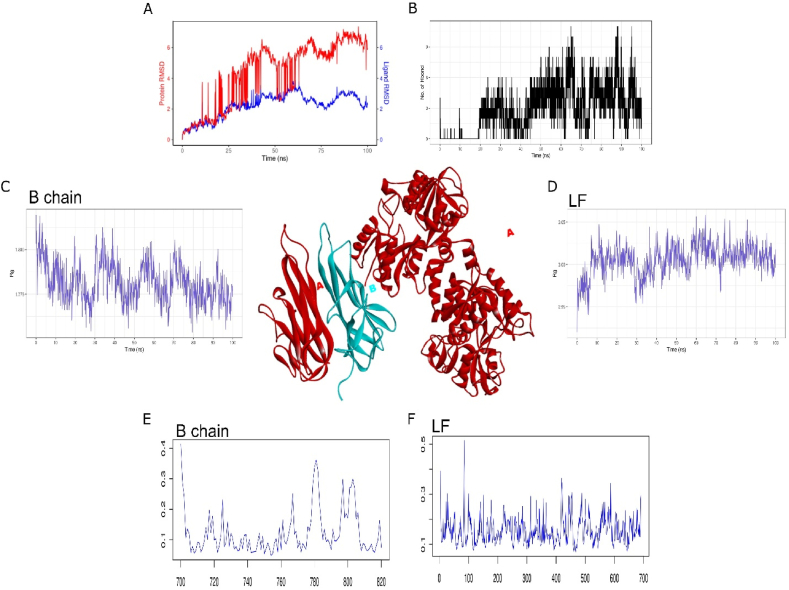
Molecular interaction of lactoferrin (in red color) with TNF-α-B chain (in blue color). Molecular dynamics simulation of 100ns between Lactoferrin and TNF-α-B chain chain used to traced A) RMSD trajectory, B) H-bond interaction, C) RoG plot of TNF-α-B chain, D) RoG plot of lactoferrin, E) RMSF plot of TNF-α-B chain (120 a.ã700–820) and F) RMSF plot of Lactoferrin (1–700 a.a). (For interpretation of the references to color in this figure legend, the reader is referred to the Web version of this article.)

Tumor necrosis factor alpha (TNF-α) is a cytokine with pleiotropic effects on various cell types. Structurally, it is a homotrimer protein consisting of 157 amino acids, mainly generated by activated macrophages, T-lymphocytes, and natural killer cells. Whether Lactoferrin is also capable of forming a stable interaction with TNF-α, we further evaluated their interaction using molecular docking and simulation. Docking and simulations of three chains have shown similar pattern of results, here we have discussed interaction of chain B in detail (Table 6). In total 9 H-bond and 10 hydrophobic interactions were observed. Atomistic interaction for the period of 100ns were further evaluated on the nature of this protein-protein interaction. RMSD plot suggests strong congruence in the start of simulation, however after 50ns the deviations have increased up to 2 Å (Fig. 7A). Rule of thumb allows the interaction to be stable if the distance between protein-ligand is less than 2 Å. Since, dynamics of protein-protein interactions are different from protein-ligand, we cannot rule out the interaction between TNF-α and lactoferrin. H-bond interaction has shown gradual increase that peaked to 9 interactions at the end of simulations (Fig. 7B). Furthermore, radius of gyration of both proteins have also shown highly compact nature of their interaction with reduced level of flexibility (Fig. 7C and D). Similarly, average RMSF of both interaction proteins have also shown reduced fluctuation with average of less than 0.4 Å (Fig. 7 E and F). In short, these interactions studies suggest lack of stable interaction between lactoferrin and TNF-α. The metanalysis on 25 selected studies have shown lower levels of TNF-α upon exposure to Lactoferrin. However significant heterogeneity (*p* = 0.001; I^2^ = 66 %) was observed [28]. Marginal level of stable protein-protein interactions and presence of data heterogeneity during in-vivo studies might suggest that lactoferrin does not have direct interaction with TNF-α. MD simulations results for both chain A and B are illustrated in Fig. S2.

**Table 6:** Molecular interactions of TNF- α with Lactoferrin.

Complex	Hydrogen Bonds	Hydrophobic Interections
TNF_LF (Chain A-B)	Glu798(B), Lys806(B), Tyr753(B), Gln755(B), Tyr813(B), Ser841(B), Gly842(B), Arg927(A), Gln926(A), Val947(A), Tyr943(A), Gly945(A), Asn916(A), Ser919(A)	Pro811(B), Leu730(B), Val707(B), Ile849(B), His709(B), Asn728(B), Pro807(B), Tyr845(B), Gln843(B), Thr896(A), Lys922(A), Gln949(A), Leu879(A), Gly946(A), Phe948(A), Leu944(A), His897(A), Leu917(A), Leu918(A)
TNFalpha_LF (chain B–A)	Gln7(A), Arg53(A), Arg4(A), Asn261(A), Asn831(B), Asp834(B), Asn786(B), Lys784(B), Glu829(B)	Ile37(A), Lys38(A), Cys36(A), Ser35(A), Val768(B), Hus767(B), Thr766(B), Leu769(B), Thr771(B), Arg832(B), Thr773(B), Ser789(B)

### 3.7 Comparison of free binding energy

The MM/GBSA analysis is widely adopted for its accurate prediction of docking scores in various molecular complexes, including protein-protein, protein-ligand, and protein-nucleic acid complexes [31]. The binding energy approach is further utilized to evaluate the binding strength in each complex. Table 7 provides the detailed profile of free binding energy and overall complex binding energy to evaluate the protein-protein interaction. IKK and LF have shown the lowest amount of free binding energy which is −247.04 kj/mol with highest contribution from H-bond and van der Waals interactions. Overall lowering the complex energy (ΔE_com_) up to −84368.90 kj/mol lowest in terms of all other complexes. Secondly, IκBα-LF have also shown contribution of electrostatic, H-bond and van der Waals interactions (ΔE_col,_ ΔE_hb_, ΔE_vdw_ respectively) with overall complex energy of −38,357 kj/mol. The overall free binding energy (ΔE_tol_) was net negative of −97.05 Kj/mol which suggest that IκBα-LF interaction was stable. IL-6 and IL-1β have also shown stable interaction with net free binding energy of −45.86 and −42.48 Kj/mol respectively. Interestingly, TNF-α have shown further lower free binding energy with highest contribution from van der Waals interactions and H-bond interactions (ΔE_tol_ = 138.75 Kj/mol). In short, the complexes which have shown relatively weaker RMSD interaction profile have shown significantly stable interaction in MMGBSA analysis. This strongly suggest that LF does not only have stable interaction with IκBα, NF-κB, 1IL-6 and IL-1β but also have strong interaction with IKK and TNF-α. All complexes NF-κB pathway have shown major contribution of both polar and non-polar interactions.

**Table 7:** MMGBSA analysis of Lactoferrin interaction with members of NF-κB pathways.

Complex	ΔE_tol_	ΔE_col_	ΔE_cov_	ΔE_hb_	ΔE_vdw_	ΔE_com_
IKK	−247.04	1327.10	8611.34	−20.99	−9294.39	−84,368.90
IκBα	−97.05	−487.48	3899.95	−15.95	−4129.51	−38,537.10
1IL-6	−45.86	198.05	3706.73	−7.91	−3854.34	−35,843.80
IL-1β	−42.48	261.60	3565.32	−5.97	−3856.60	−36,039.50
TNF-α	−138.75	374.56	4542.97	−11.67	−4468.59	−41,680.00

## 4 Discussion

Lactoferrin, while not inherently immunogenic, plays a pivotal role in modulating both innate and adaptive immune responses [4]. Its antimicrobial properties contribute to its immunomodulatory effects, acting as a chemo-attractant for immune cells and modulating their functions [5]. Additionally, lactoferrin demonstrates antiviral properties, affecting T-cell and B-cell responses, and is recognized by the immune system through its receptors. The immunomodulatory effects of lactoferrin and its derived peptides have been extensively studied, positioning it as a first-line defense protein against various microbial infections [5].

Recent meta-analyses have indicated that lactoferrin modulates proteins involved in the downstream signaling pathway of NF-κB [28]. This study provides compelling evidence that LF interacts stably with IL-1β, IL-6, IκBα, and NF-κB, and relatively weaker interactions with IKK and TNF-α. These interactions were confirmed through various molecular dynamics simulations, including RMSD, RMSF, hydrogen bond interactions, and radius of gyration (RoG) analyses. The MM/GBSA analysis further supports the stability of these interactions, indicating that LF has the lowest free binding energy with IKK, followed by IκBα, IL-6, and IL-1β. This suggests strong and stable interactions, particularly with IKK and IκBα.

The study highlights LF's potential to modulate inflammation and improve insulin sensitivity by interacting with the NF-κB pathway. LF's interaction with NF-κB pathway components suggests it can inhibit the pathway's activation, reducing the production of pro-inflammatory cytokines such as TNF-α, IL-1β, and IL-6. By stabilizing IκBα and preventing NF-κB translocation to the nucleus, LF can effectively downregulate inflammatory responses. This modulation can enhance insulin signaling and glucose uptake by cells, thereby improving insulin sensitivity and aiding in diabetes management. The study also suggests that LF and its peptides could be developed as preventive and therapeutic agents targeting the NF-κB pathway to manage diabetes-associated inflammation. These findings align with *in-vivo* consensus, where high-dose lactoferrin exposure reduced TNF-α, while lower doses were sufficient to inhibit the phosphorylation of IκBα [28]. In summary, our study offers groundbreaking evidence of the direct interaction of lactoferrin with the six major components of the NF-κB pathway, consistent with *in-vivo* findings.

LF's anti-inflammatory properties are well-documented. It has been shown to reduce the levels of pro-inflammatory cytokines such as IL-1β, IL-6, and TNF-α, which are associated with insulin resistance and chronic inflammation in diabetes [32]. LF's ability to modulate the NF-κB pathway and its downstream signaling components, such as PPAR-γ and SIRT-1, further supports its role in reducing inflammation and improving metabolic health [33]. Moreover, *in-vitro* and *in-vivo* studies have shown that LF improves insulin sensitivity even in the presence of existing insulin resistance, reduces weight gain, and regulates blood sugar levels, leptin, and lipid levels in animal models [32]. Additionally, LF has been found to improve hepatic insulin resistance and pancreatic dysfunction in high-fat diet and streptozotocin-induced diabetic mice [34]. Clinical studies have also demonstrated the antidiabetic effects of LF, showing significant improvements in HbA1c, body mass index, and lipid status in pediatric patients with type 2 diabetes. Additionally, LF has been found to improve hepatic insulin resistance and pancreatic dysfunction in high-fat diet and streptozotocin-induced diabetic mice by regulating the PI3K/AKT signaling pathway. Clinical studies have also demonstrated the antidiabetic effects of LF, showing significant improvements in HbA1c, body mass index, and lipid status in pediatric patients with type 2 diabetes [33].

In short, the findings of this study provide evidence that Lactoferrin have shown direct interaction with components of NF-κB pathway which could be responsible for downregulation of pro-inflammatory components. These findings are crucial for the development of novel therapeutic interventions targeting inflammation in diabetes. Both N and C lobe have shown inhibitory properties for downregulating pro-inflammatory proteins. Targeted peptides from each lobe can be used for development of drugs or treatments aimed at mimicking its beneficial effects. This could lead to more personalized and effective management strategies for diabetes, considering the heterogeneity of inflammatory responses among patients. Lactoferrin derived peptide-based drugs will specifically target NF-κB pathway modulation which will have fewer sides effect compared to broad spectrum anti-inflammatory drugs. This could enhance patient compliance and reduce the risk of adverse reaction. Targeted inhibition of NF-κB pathway can ultimately contribute to improve treatment regimens and achieving better glycemic control. This approach is also used for the development of preventive strategies for individuals at risk of developing diabetes. A proactive approach may help to mitigate the onset and progression of diabetes, potentially reducing the burden on health care system and improving public health outcomes.

## 5 Conclusion

In conclusion, lactoferrin emerges as a promising agent in the management of type 2 diabetes through its interaction with the NF-κB pathway. Our study provides compelling evidence through molecular docking and simulation approaches that lactoferrin interacts with key components of the NF-κB pathway, including IL-1β, IL-6, IκBα, and NF-κB. These stable interactions suggest a potential for lactoferrin to modulate inflammation and improve insulin sensitivity, offering avenues for preventive and therapeutic interventions in diabetes. Our findings pave the way for further research elucidating the precise mechanisms and clinical applications of lactoferrin and its peptides in diabetes management.

## Funding

No funding was received to execute this research.

## Availability of data and materials

All data generated and analyzed during this study are included in this article along with supplementary files. The data and materials used in this study are available upon reasonable requests to the corresponding author.

## Ethics approval and consent to participate

This article does not require ethical approval because there are no human and animal participants.

## Consent for publication

Not applicable.

## Ethics approval

This article does not require ethical approval because there are no human and animal participants.

## CRediT authorship contribution statement

**Muhammad Asif Arain:** Writing – original draft, Software, Data curation, Conceptualization. **Gul Bahar Khaskheli:** Writing – review & editing, Supervision, Resources, Methodology. **Ghulam Shabir Barham:** Writing – review & editing, Validation, Formal analysis. **Illahi Bakhsh Marghazani:** Writing – review & editing, Validation, Software.

## Declaration of competing interest

The authors of this manuscript declared that they have no potential conflict of interest.

## Figures and Tables

**figs1:**
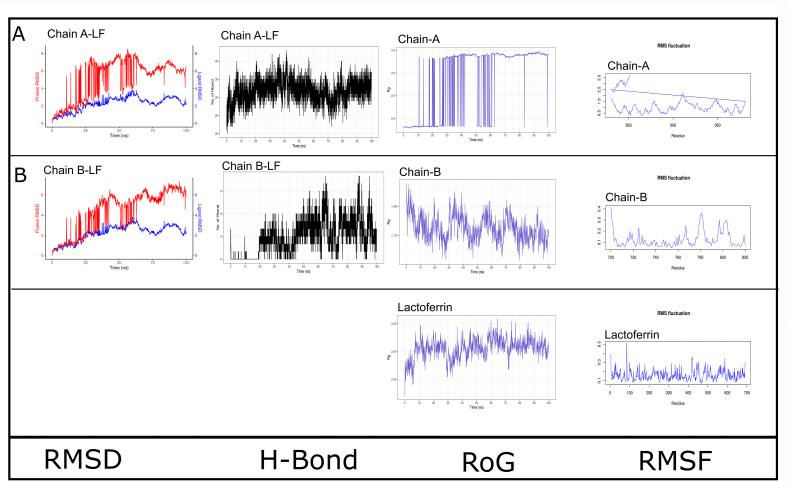


**figs2:**